# The Effects of Proprioceptive Training on Balance, Strength, Agility and Dribbling in Adolescent Male Soccer Players

**DOI:** 10.3390/ijerph19042028

**Published:** 2022-02-11

**Authors:** Diana Victoria Gidu, Dana Badau, Marius Stoica, Adrian Aron, George Focan, Dan Monea, Alina Mihaela Stoica, Nicoleta Daniela Calota

**Affiliations:** 1 Faculty of Physical Education and Sport, “Ovidius” University from Constanta, 900470 Constanta, Romania; campiap@yahoo.com (D.V.G.); nicocalota@yahoo.com (N.D.C.); 2 Petru Maior Faculty of Sciences and Letters, George Emil Palade University of Medicine, Pharmacy, Sciences and Technology, 540142 Targu Mures, Romania; 3 Interdisciplinary Doctoral School, Faculty of Physical Education and Mountain Sports, Transilvania University, 500068 Brasov, Romania; 4 Faculty of Physical Education and Sports, National University of Physical Education and Sports, 060057 Bucharest, Romania; 5 Department of Physical Therapy, Radford University, Roanoke, VA 24013, USA; aaron@radford.edu; 6 “Farul” Academy Constanta, 900635 Constanta, Romania; georgefocan@yahoo.com; 7 Faculty of Physical Education and Sports, Babes Bolyai University, 400048 Cluj-Napoca, Romania; moneadan@yahoo.com; 8 Department of Physical Education and Sport, University of Bucharest, 050107 Bucharest, Romania; alina.stoica@g.unibuc.ro

**Keywords:** proprioceptive training, physical fitness components, soccer skills, balance, strength, agility

## Abstract

The aim of the study was to determine the effects of proprioceptive training (PT) on balance, strength, agility and dribbling in adolescent soccer players. In this research, we included an experimental (*n* = 48) and a control (*n* = 48) group (CG) with 14 years old players. The experimental group (EG) participated in an 8 week PT program, with four 30 min sessions per week. The experimental program included 12 bosu ball exercises to improve balance, stability and strength which were grouped into two subprograms: the first not using the soccer ball, the second subprogram using the soccer ball. The subprograms were implemented alternately during 16 proprioceptive training sessions, on two types of firm and foam surfaces. Pre- and post-tests included the static balance [Balance Error Scoring System (BESS)], vertical, horizontal, and lateral jumping, and the completion of agility (“arrowhead”) and dribbling (“short dribbling”) tests. Regarding the total BESS score, the CG has demonstrated progress between the pre- and the post-test, with 0.780 ± 0.895, fewer errors, while the EG had 5.828 ± 1.017 fewer errors. The difference between the two groups was of 5.148 fewer errors for the EG who had practiced the proposed program of proprioceptive training. The highest difference registered between the pre- and the post-test was at the test “single-leg forward jump with the right leg”, with a result of 1.083 ± 0.459 cm for the CG and of 3.916 ± 0. 761 cm for the EG. Through the analysis of average differences between the pre- and the post-tests, we observe that, regarding the “Agility right side test”, the EG has progressed with 0.382 s in comparison with the CG; regarding the “Agility left side test”, the EG has progressed with 0.233 s compared to the CG; regarding the “Agility right and left side test”, the EG has progressed with 0.196 s compared to the CG; in the “Short dribbling test”, the EG has progressed with 0.174 s compared to the CG. The highest progress was made at the “Agility right side test”, of 0.402 s for the EG, while the CG registered 0.120 s. Most of the results in all tests for both experimental groups show an effect size ranging from small to medium. The progress made by the experimental group in all tests was statistically significant, while in the control group the progress was mostly statistically insignificant for *p* < 0.05. The results suggest that a PT program performed at about 14 years of age could be successfully implemented in the training regime of soccer players to improve components of fitness along with dribbling skills. The results of the study revealed that sports training on the foam surfaces determined a superior progress of the development of proprioception compared to the increased training on the firm surfaces.

## 1. Introduction

Proprioception is the reception of stimuli produced within an organism and it refers to the conscious and unconscious perception of postural balance, muscle sense and joint stability [1,2]. Proprioception regards the human ability to perceive the position of the body, the speed of movement and the general or specific resistance [1,2,3]. Because of its involvement in joint stability and injury prevention, proprioception plays a very important role in sport. Proprioceptive or balance training (PT) [4], also called sensorimotor training [5,6], was originally proposed by clinicians as one of the rehabilitative exercise concepts [7]. To date, a large number of simple or more complex, static or dynamic exercises were proposed as proprioceptive, making it difficult to choose between them when intending to design an intervention program. However, in the opinion of Riva et al. [4], such exercises should require the management of instability, even if is obvious that different movements and postures classified as proprioceptive by this criterion will generate different levels of proprioceptive involvement and effectiveness.

When speaking about the benefits of PT for neuromuscular control, we refer to improvements in muscle reflex activity [6,7,8,9,10,11], reaction time [12]), rate of force development and electromiography activity [13]. On the other hand, the outcomes of interest for functional performance are expressed in terms of postural control [14,15], agility [15,16], muscle strength [15], jump performance [13,16,17], and sprint time [16,17,18].

According to Zech et al. [19], the PT improvements in balance skills are clearly documented, compared to the effects on jump, sprint, agility and strength, or neuromuscular outcomes, that appear inconsistent. Apart from the large variability of exercises, the explanation of this incongruence lies in the characteristics of the subjects and the dosage of balance training [9,10,14,16]. Specifically, the training status of subjects seems to be relevant; for instance, PT had no effects on strength in athletes and recreationally active persons, but significantly improved knee muscle strength in nonathletes [19,20,21,22,23].

It is accepted that age and sport disciplines could have an impact on proprioceptive performance [24,25,26,27,28,29]. PT was traditionally used to improve fitness components in both athletes and nonathletes, however, in the last years several studies on healthy athletes investigated its effects on specific sports skills. For instance, Kostopoulos et al. [30], showed that PT improves the basketball players’ passing skills, while young female handball players may score more goals when balance improvements are present [31]. Few other studies have drawn attention to the possible effects on some specific soccer performances [32,33,34,35]. Research has shown a key influence of PT on the static and dynamic balance especially in individual sports, contact sports, and sport games [36,37,38]. However, a problem with these studies is that they only investigated the PT effects on specific sport skills [30,32,33], and not in relation to the impact of multiple concomitant fitness components.

The assessment of the PT impact on the development of balance, explosive strength, agility and dribbling performance has been under investigated for the age group of 14 years old soccer players. We believe that our study contributes to the broadening of knowledge regarding the development of physical fitness components through a PT program tailored for the particularities of both the physical and technical preparation of the soccer game, and the players’ age. An innovative trait of this study consists of the assessment of how two surface types (firm vs. foam) affect the development of balance, of the sense of touch and the decision-making capacity of junior soccer players.

The hypothesis of the study was based on the assumption that by designing and implementing an experimental proprioceptive program that will be applied to two types of surfaces: firm and foam, will contribute to improving balance, strength, agility, and dribbling performance in young soccer players. Therefore, the aim of the present investigation was to evaluate the effectiveness of PT on balance, strength, agility, and dribbling performance in young soccer players and to assess how the type of surface influences the players’ static balance.

## 2. Materials and Methods

### 2.1. Participants

For this research we included 96 male soccer players of a team competing in the second junior league. The subjects were divided into a control group of 48 players (M ± SD: age 14.0 ± 0.0 years; height 170.7 ± 6.4 cm; body mass 61.1 ± 5.4 kg; percent fat 15.3 ± 3.0%, 96% with right foot dominance and 4% with left foot dominance) and an experimental group of 48 players (age 14.2 ± 0.4 years; height 169.0. ± 9.1 cm; body mass 58.7 ± 11.1 kg; percent of fat 13.6 ± 4.9, 94% with right foot dominance and 6% with left foot dominance.). The podal dominance was considered the foot with which the subjects of the study hit the ball with increased force and precision. The subjects were assigned to groups based on their practice schedule, thus creating a control group that practiced mostly in the morning.

The criteria of selection were: the participants had to be 14 years old, to practice soccer actively, to have the physical abilities required and medical consent to play soccer, to have completed the proprioceptive training program, to take all the tests required in the study. The criteria of exclusion from participating to the study were: poor health and incomplete participation in the training and testing program.

### 2.2. Design and the Procedure of Research

The study had the following structure: between 8 and 9 April 2021, the pre-testing of all the subjects was accomplished, from 12 April to 4 June 2021 (8 weeks) the proprioceptive training programme was implemented to the experimental group (EG) only, while between 7 and 8 June 2021 the post-testing of all the participants in both the EG and the control group (CG) was carried out. The order of the tests was similar, both before and after the intervention: Balance Error Scoring System (BESS), single-leg lateral and horizontal (forward) jumps and arrowhead test (AT) on the first day, and single-leg vertical jumps, double-leg countermovement jump (CMJ) and short dribbling test (SDT) on the second day.

The proprioceptive training program was implemented only in the EG, and it included exercises tailored according to the characteristics of soccer game and of the participants’ age, while the CG followed a training program aiming the development of effort capacity through athletic means and soccer–specific techniques. To evaluate the effects of a PT program on the balance, strength, agility, and dribbling performance a quasi-experimental design was used. The intervention, with four PT sessions per week, has been carried out during the competition phase of the training year. During the 8-week PT, the total training time per day and per week was the same for both groups, but while the control group (CG) simply performed their normal program of that period, the experiment group (EG) engaged in four 30 minutes’ proprioceptive exercises per week.

For this study, including the training and evaluation processes, we selected two types of surfaces, one firm and the other foam. The firm surface is a dry natural surface with short cut grass that gives a feeling of hardness in podal contact. Foam surfaces model Astro-Turf, artificial foam and short grass surfaces that give a soft feeling in podal contact. The two surfaces require different proprioceptive adaptations in order to adapt to the characteristics of the surfaces in the case of podal contact as is soccer-specific.

The study was approved by the club’s manager and performed according to the principles expressed in the Declaration of Helsinki. Written informed consent was obtained from the parents, while the children signed informed assents. All authors contributed equally for this article; all authors have an equal contribution with the first author, too. This research was approved on 22 January 2021 by the Review Board of the Physical Education and Sports Department from Ovidius University of Constanta.

### 2.3. The Proprioceptive Training (PT) Program

Taken together, 12 exercises were included in the PT programme; all of them were performed using Both Sides Utilized Balance Trainer (BOSU^®^). They were divided into two subprogrammes; the first subprogramme included six exercises that did not involve ball usage, whereas those in the second subprogramme were performed with the soccer ball. In both cases, the first three exercises were intended to improve balance and stability, the rest aiming at influencing balance and strength. The two subprogrammes were continuously alternated from session to session, for a total of 16 sessions for each one. The PT program was performed during the usual warm-up, in such a way that both groups had a similar amount of total training. When an exercise was performed on a single leg, the legs were continuously alternated within a set. Wearing firm soccer boots, the subjects performed the programme on a familiar natural grass field. For accommodation with the PT content, the EG subjects performed a set of repetitions for each exercise, the day before the first session of intervention, after the dribbling test.

Proprioceptive training program:

- Balance training subprogram 1–without soccer ball.
  For balance and stability, we used three exercises:
  − Squats on both legs standing on the bosu ball; 4 × 10, time recovery 30 s.
  − Squats on one leg standing on the bosu ball; 4 × 10 per limb, time recovery 30 s.
  − Swinging leg forward, backward, and lateral while standing on one foot on the bosu ball; 4 × 10 per limb, time recovery 30 s.
  − For balance and strength, we used three exercises:
  − Forward jumps on one foot, from the ground on the bosu ball, and holding the landing position for 2–3 s; 4 × 10 per limb, time recovery 30 s.
  − Lateral jumps on one foot, from the ground on the bosu ball, and holding the landing position for 2–3 s; 4 × 10 per limb, time recovery 30 s.
  − Forward jump lunge on two bosu ball; 4 × 10, time recovery 30 s.
- Balance training subprogram 2–with soccer ball.
  For balance and stability, we used three exercises:
  − Kicking a soccer ball thrown by a team-mate while standing with both feet on bosu ball; 4 × 10 per limb, time recovery 30 s.
  − Kicking a soccer ball thrown by a team-mate while standing on one foot on bosu ball; 4 × 10 per limb, time recovery 30 s.
  − Heading a soccer ball thrown by a team-mate while standing on one foot on bosu ball; 4 × 10 per limb, time recovery 30 s.
  − For balance and strength, we used three exercises:
  − Forward jumps from a bosu ball to another, holding the landing position for 2–3 s, while kicking a soccer ball thrown by a team-mate; 4 × 10 per limb, time recovery 30 s.;
  − Kicking a soccer ball thrown by a team-mate while standing on one foot on the bosu ball with an elastic band tied around both feet; 4 × 10 per limb, time recovery 30 s.;
  − Move the soccer ball around the bosu ball while standing on one foot on the bosu ball; 4 × 6 per limb, time recovery 30 s.

### 2.4. Measures

To investigate the effects of PT, Balance Error Scoring System (BESS) was used to test postural stability, single- and double-leg jumps for explosive strength, arrowhead test (AT) for agility, and short dribbling test (SDT) for the coordination and speed with the ball. While most of the studies on PT used instrumental methods to assess balance, very few of them took advantage of BESS [39]. We used BESS because of its lack of complexity, high sensitivity [40], moderate to good reliability and construct validity, and correlation with laboratory-based measures for criterion-related validity [41]. Both hard and foam surfaces tasks of the BESS test were significantly correlated with forceplate sway [41], demonstrating the relevance in soccer, where the single leg dynamic instead of static balance is involved. Although some studies in soccer players only estimated total score [35], or just single leg stance for each limb [24], our subjects were evaluated on double leg and tandem, and also on single nondominant leg condition. We tested only the nondominant leg because, in most players, it is the leg of support and taking off, and is considered of interest to estimate the training impact on its balance competences. For the BESS testing, the barefooted subjects were asked to perform two times each of the three variants of BESS test–double, single (only nondominant), and tandem leg support, on firm and Astro-Turf foam surface, respectively. We mention that the Astro-Turf foam surface is certified by FIFA, and the company surface has been verified and validated by the experts of the Romanian Football Federation. The starting and reference position was akimbo with closed eyes. The 20-s observation period started at closing eyes and the errors were defined according to Bell et al. [41].

Considering the high frequency and special importance of jumps in different forms and planes in soccer, for a comprehensive evaluation of our subjects we tested their explosive strength with both single-leg lateral, horizontal (forward) and vertical jumps, and double-leg countermovement (CMJ) jump. Within all the jumps, the subjects were asked to keep hands on the hips, to sink to a self-selected depth as quickly as possible, and then jump as far or high as they could, landing on the same leg in single-leg lateral and horizontal jumps and on both legs in vertical jumps. The distance jumped in single-leg horizontal and lateral tests was measured with a tape, whereas the heights were displayed by a contact mat system of the MobileMatTM BESS system. For each jump, three attempts were performed, with a 2 minutes’ rest, and the best result was considered for analysis.

The Arrowhead Test (AT) [42], was selected to assess agility. The application of the Arrowhead Test required the following equipment: timing gates Microgate, measuring tape, six marking cones, a flat abrasive surface. Out of the several tests validated, this was preferred because it allows the comparison of players’ speed, explosion, and body control when they are asked to predominantly change the direction to the right, and to the left. These comparisons were further helpful in choosing the appropriate drills needed to be performed in practice. The AT consisted of one trial to the left and one trial to the right, separated by 6–7 min of recovery. It was performed two times, with the best result recorded for analysis.

Dribbling performance was assessed using the short dribbling test (SDT) proposed by Bangsbo & Mohr [42]. It is considered that completing an agility test without a ball is not sufficient to characterize the players’ specific performance capability in a sport where dribbling is a key skill for success. In addition, there is limited research that investigates if and/or how PT influences the dribbling skill. To familiarize subjects with the tasks, in the case of both AT and SDT, before the real testing, they completed the test course two times: at a self-selected low and moderate pace. Then, each test was performed two times and the best result displayed by Microgate electronic timing equipment was considered for analysis. A standardized ten-minute warm-up protocol was used before all the measurements.

### 2.5. Statistical Analysis

Statistical analyses were performed using SPSS 22 and significance was set to *p* < 0.05. For all the results of the study, for pre- and post-test, for the right/left leg or for the executions with both legs we calculated: X ± DS-mean ± standard deviation, and to identify the differences between the testers we calculated the statistical parameters: DX– difference of means, 95% CI Lower/Upper-confidence interval with two levels lower and upper, Student’s *t*-test, p-statistical lever of probability, d-Cohen ‘d effect size. The interpretation of the Cohen ‘d effect size was: 0.1–0.2 small, 0.3–0.5 medium, 0.5–0.8 large, over 0.8 very large [43]. Multiple linear regression analysis was performed to determine the amount of influence which the various tasks of the BESS had on the total BESS score. The normality distribution for this study was calculated with a Shapiro–Wilk test. For reliability we calculated the coefficient of variation (CV), for pre- and post- test reliability we used Intraclass correlation coefficient (ICC) with 95% CI. In order to determine the differences between the progress of the three independent groups, we used the Wilcoxon, calculating the parameter Z and its significance.

## 3. Results

The analysis of mean differences (Table 1) registered between the pre- and the post-testing for the “Single-leg stance” test highlights that the EG has made a progress in comparison to the CG, with 0.52 less errors on firm surface, respectively with 1.623 less errors on foam surface. At the “Double-leg stance” test, the EG has progressed compared to the CG, with 0.253 errors on firm surface, respectively with 1.176 less errors on foam surface. At the tandem stance test, the EG has progressed compared to the CG, with 0.183 fewer errors on the firm surface, and with 0.712 fewer errors on the foam surface.

The progress made by the CG for all tests was statistically irrelevant, while the progress of the EG was statistically relevant, where *p* < 0.05. The Cohen ‘d effect size analysis shows that the results of the CG in all the BESS tests were under 0.3, which indicates a small effect, while for the EG, all the results in all the tests were between 0.5 and 0.8 which is classified as a medium effect. The analysis of the results of balance performance test showed a normal distribution of the results which ranged between 0.709–0.824. The values of the coefficient of variation for the control groups at the balance performance tests were between 12–15% which reflects an average reliability, while the experiment group recorded values between 4–9% which reflects low dispersion, so a high reliability. For all tests of the balance performances, ICC values (95%CI) were between 0.402–0.524 which showed a medium reliability. Regarding the total BESS score, the CG has made a progress of 0.780 fewer errors between the pre- and the post-testing, while the EG had 5.828 fewer errors. The difference between the two groups of 5.148 fewer errors for the EG, who had practiced the proprioceptive training program proposed. The results highlight that both groups had a higher number of errors on the foam surface compared to the firm surface, which reveals that the more unstable the surface is, the more involved are the capacity of balance and the sense of postural control. The Wilcoxon test analysis showed statistically significant differences between the averages of the experimental group and the control group for the four motor tests, as follows: for Single-leg forward jump test Z −4.342, *p* = 0.002 for firm surface and Z −3.342, *p* = 0.000 for foam surface; for Double-leg stance test Z −4.342, *p* = 0.002 for firm surface and Z −3.342, *p* = 0.000 for foam surface; for Tandem stance test Z −4.121, *p* = 0.000 for firm surface and Z −2.935, *p* = 0.000 for foam surface; for BESS score Z −2.738, *p* = 0.000.

Using a multiple regression analysis of the score changes in the EG, we calculated that single leg stance on foam surface explained 73% of the variance in the total BESS score (R^2^ = 0.73; *p* = 0.0003). Tandem stance on foam accounted for just 17% of the variance (R^2^ = 0.17; *p* = 0.003), and was followed by single leg and tandem stance on firm surface, both at 5% (R^2^ = 0.05; *p* = 0.04 and 0.02, respectively). Worth observing is that although single leg stance on foam was the hardest condition, it displayed the greatest reduction in errors (from 3.66 to 1.66, which means an average decrement of 2 errors, i.e., 54.64%), while accounting for nearly ¾ of the total score improvement.

As shown in Table 2, the results of the jump tests show that both groups had better scores for the right leg compared to the left one. This result is due to the higher number of junior soccer players participating in the study, who had right-leg dominance. The progress made by the CG was statistically irrelevant for all the tests, while the progress of the EG was statistically significant, where *p* < 0.05. The Cohen ‘d effect size analysis shows that the results of the CG, for the accomplishments with the right leg and with the left leg, in all the BESS tests indicated a small effect, meanwhile, the results of the EG in all the tests indicated a medium effect. The analysis of balance performance results showed a normal distribution of the results which ranged between 0.732–0.863. For the jump performance tests, the values of the coefficient of variability showed a medium dispersion for the control group and low dispersion for the experimental group. The interclass correlation coefficient for both groups showed an average reliability.

It is important to note that the accomplishments of the EG have been superior to those of the CG, in all the tests, for both right-leg and left-leg performances (Table 2). In the test Single-leg forward jump, the mean differences registered between the two groups were 2.833 cm for right-leg executions, and 1.831 cm for left-leg executions. In both cases, the performance of the EG was superior to that of the other group. In the test Single-leg lateral jump, the mean differences between the two groups were also in favor of the EG, with 2.604 cm for right-leg executions and 2.208 cm for left-leg executions. In the test Single-leg vertical jump, the comparative analysis of the arithmetic means registered between the two groups showed that the progress of the EG was superior compared to that of the CG, with 1.048 cm for the right-leg test and 0.717 cm for the left-leg test. In the test Double-leg CMJ, the mean differences registered between the two groups were 1.259 cm for the EG. The highest differences registered between the pre- and the post-test was in the test Single-leg forward jump with the right-leg, with 1.083 cm for the CG and 3.916 cm for the EG, while the lowest differences were obtained in the test Single-leg vertical jump with the left-leg, with 0.247 cm for the CG and 0.964 cm for the EG. The differences between the groups were statistically significant for all jump tests highlighted by the Z values of the Wilcoxon test which were: for Single-leg forward jump right/left Z −14.831/−14.261, *p* = 0.000; for Single-leg lateral jump right/left Z −9.923/−8.124, *p* = 0.000; for Single-leg vertical jump right/left Z −8.942/−9.164, *p* = 0.000/001; for Double-leg CMJ Z −11.462/9.946, *p* = 0.010.

Analyzing the mean differences between the pre- and the post-tests, we observe that, regarding the Agility right side test, the EG improved with 0.382 s compared to the CG; regarding the Agility left side test, the EG improved with 0.233 s compared to the CG; regarding the Agility right + left side test, the EG improved with 0.196 s. compared to the CG, while for the Short dribbling test, the EG improved with 0.174 s. compared to the CG. The highest improvement was obtained for the Agility right side test, of 0.402 s. for the EG, and 0.120 s. for the CG. For the agility and dribbling tests, the differences between the two groups of the study were statistically significant, all values of the Z parameter having the value of the significance threshold *p* < 0.05.

In this section of the study, only EG improvements were statistically relevant. The Cohen ‘d effect size analysis reveals that the results of the CG in all the agility and dribbling tests were below 0.3, which indicates a small effect, while the results of the EG in all the tests were between 0.5 and 0.8, which fit into the medium category. All the arithmetic means, for all the tests of both groups, were between the lower and the upper limit of 95% CI (Table 3). The distribution of the data was normal in the agility and dribbling tests, falling between 0.763–0.897. The result of the agility and dribbling test showed a low dispersion, meaning a very good reliability for the experimental group, meanwhile, the control group showed a medium reliability. The values of ICC for 95%CI for both groups of the study showed an average rehabilitation for all tests of agility and dribbling.

## 4. Discussion

The objectives of this study were: to investigate whether a PT program simultaneously influences balance, strength, agility and a sport specific skill (dribbling) in young soccer players, and also to assess which types of surfaces influence static balance. The results of our study reveal an improvement in the accomplishments of the EG compared to the CG regarding balance, strength, agility and a sport specific skill (dribbling) in young soccer players as a result of the implementation of the PT program. The findings presented in our study fill in a knowledge gap regarding this specific topic and, considering the superior accomplishments of the EG compared to the CG we can conclude that the implementation of the PT program has contributed to a significant improvement of the physical fitness of the junior soccer players included in the EG. Moreover, the results obtained by measuring the level of balance and the sense of posture on the two types of surfaces with different elastic properties showed that foam surfaces are much more efficient compared to firm surfaces in order to optimize the components of proprioception. We believe that the findings of this study complement the conclusions of recent research dealing with the improvement of physical fitness by execution of specifically-tailored proprioceptive programs.

Using a multiple regression analysis of the score changes in the EG, we calculated that single leg stance on foam surface explained 73% of the variance in the total BESS score (R^2^ = 0.73; *p* = 0.0003). Tandem stance on foam accounted for just 17% of the variance (R^2^ = 0.17; *p* = 0.003), and was followed by single leg and tandem stance on firm surface, both at 5% (R^2^ = 0.05; *p* = 0.04 and 0.02, respectively). The results of the study underline no significant decrease in errors in single leg firm surface stance contributing to the total score improvement. Worth observing is that although single leg stance on foam was the hardest condition, it displayed the greatest reduction of errors, while accounting for nearly three quarters of the total score improvement. One plausible explanation can be attributed to the single leg exercises of the program and especially to those of the second subprogram. Thus, unlike McLeod et al. [44], we found that PT decreased errors not only in single and tandem on foam surface but also in tandem on firm surface condition.

The main mechanism of BESS improvement after intervention could stem from the process of learning to better pay attention to biomechanical cues, for instance to joint acceleration; which, in turn, further improves the probability to detect any little change in joint position [45]. Other mechanisms may be related to the improvements in neuromuscular coordination and joint range of motion (ROM), or simply to the better joint strength [24], also seen in our experiment.

### 4.1. Strength Effects

The interest in the possible beneficial effect of PT on strength seems to have begun a long time ago, when it was found that the maximal isometric force of the knee and ankle muscles increased after a wobble board program [17]. In a study by Heitkamp et al. [46], it was concluded that PT and strength training generate similar improvement in maximal isometric force of knee muscles. In the following years it was shown that PT may increase one or more of the strength parameters–one-repetition max, maximal voluntary contraction, maximal rate force development, rate force development in various or in all time intervals of maximal voluntary contraction, peak isometric/isokinetic torque, and rectus femoris reactive activity. These improvements can be seen in sedentary [47], non-specified, rather sedentary [5], recreationally active [13], or trained individuals [16]. Other studies found no effects of PT on maximum isometric force and maximal rate of force development in athletes [48], or on isokinetic peak torque, in recreationally active subjects [15,16].

Apart from the mentioned parameters, a different type of jumps was assessed in relation to PT, the double leg CMJ being the most often used [6,15,16,17,48,49,50,51]. Other types of jumps–horizontal/forward [17,18,50], lateral/side [17,18], vertical or double-leg horizontal [50], squat [6,52] and drop [6,16,17] were not as frequently investigated. In our study, the vertical jump improved in both double-leg CMJ and single-leg trials. PT has been shown to impact the right and not the left single leg vertical jump [50], as well as the CMJ in male athletes [6,48], in physically active subjects at the end of adolescence (i.e., about 19 years) [50], in adult recreationally active women of 25.2 years [13], and in middle aged (56.0 years) sedentary subjects of both sexes [47]. The improvements from PT are also evidenced when using a double-leg squat jump test [6,52]. There are also studies that did not find any beneficial effect of PT on double-leg vertical jump [16,49,53], which can mainly be explained by their subjects: elite athletes [16,49], or children under 7 years old, with immaturity of the postural control system and deficits in attentional focus during practice [49,53].

To better evaluate the degree of improvements, our subjects were also evaluated on jumps in the sagittal and frontal plane. Following PT, the subjects had a significant improvement on both legs in forward (*p* < 0.01), but only on the right one (*p* < 0.001) in lateral jump. The lack of improvement in the left leg could be related to the left limb support and taking-off role of the right-footed soccer players, because when the initial strength status is high, the improvements after PT may be minimal or absent [53]. This reasoning could be valid if we analyze the initial performances displayed by the groups. The EG’s initial single-leg lateral jump on left limb was trending 5 cm longer, possible because only one of the EG subjects was left-footed compared to three in the CG.

Of previous studies, only one [17] addressed the PT impact on single-leg forward and lateral, while another [50] investigated just the effects on lateral jump. In elite women soccer players both jumps showed improvement, in both right and left leg [17], while in non-athletes, PT created a change only for one side, on the right (dominant) leg [50]. Another study investigating single-leg forward and lateral jumps, did not refer to isolated performances, and mentioned just the lower limb asymmetry [18]. However, as the asymmetry significantly diminished, one can assume that the PT generated indeed some alterations in the performances of at least one leg.

Although some distinctions in the activated muscle groups and their actual level of involvement were documented [17,50], it is expected that the mechanisms through which PT influences the various jumps have many aspects in common. The better jumps of our EG group could most likely be explained by an improved intra- and intermuscular coordination of the lower leg extensor muscles [7].

Furthermore, improvements in balance may have decreased the proportion of prime mover muscles allocated to stabilization, allowing them to contribute in a greater measure to the propulsion of the body [17]. In fact, Anderson and Behm [54] demonstrated that an individual with an unstable base may not concentrate all their propulsive strength in the optimal direction. Additionally, it was suggested that even 4 weeks of PT may increase the rate of explosive strength development of the leg extensors, as a consequence of the enhanced reflex contribution acting on the spinal level [5], and withdrawal of presynaptic inhibition of the terminals of the primary afferent fibers (Ia) which has a determined role in stretching and monitoring the speed with which the muscle spindle changes [48].

### 4.2. Agility Effects

Following the PT, the two components of the Arrowhead Test did not change, however, the total time improved. Changing direction of running along with starting and stopping quickly are very common and important skills in sport. However, to the best of our knowledge only a few studies investigated PT effect on agility to date. In five of these studies, field tests were administered, while one laboratory study measured reaction time on so called simple- and multi-choice agility tasks [53]. The field tests used were the *t*-test [16], the 20 yards-run (for forward agility), side steps (for lateral agility), side jumps over the bench during 10 s [50], and shuttle run [15,16,17]. None of these field tests proved to be the most reliable or valid for measuring agility [55]. As no previous study used AT, the only possible comparison was against the test’s normative data [42]. Our subjects tested their agility within normal limits, or even seem to be superior considering that the worst performance (17.1 ± 0.5 s), initially displayed by the control group, was still 0.3 s better than that of elite Danish players of the same age.

This improvement is consistent, albeit smaller than those communicated in previous studies [16,17] in which the time obtained was nearer than that displayed by our subjects. The specificity of soccer playing and training, within which changing direction, stopping and explosive starting occur so frequently, may explain our minimal improvement. An already heightened physical quality does not allow too much room for improvement. This is evidenced by studies where adult elite soccer players have the smallest improvements among other athletes, after an intervention even longer than ours (10 weeks of PT) [17]). Why this small improvement was still significant in our study, could be a consequence of the very high homogeneity the EG group displayed in both initial testing session (SD = 0.68) and in the direction of performance modification after intervention, taking into consideration that absolutely all 12 subjects improved their time, with performances between 0.01 and 0.09 s.

As in the case of other dynamic tasks, improvements in agility may be attributed to neurological adaptations [15,16,17]. However, because of its complexity and diverse manifestations, several other factors seem to make contributions to the agility expression. A recent study confirmed that agility is significantly correlated with speed in both genders, with power in women and with balance in men [55]. Balance improvement after PT can further impact complex movements and could result in improved agility, as the AT contains stops, changes of direction and accelerations. When stopping, the improved balance ensures a better stability of the body, counteracting the inertia and preventing the body segments to continue moving in the previous direction. This facilitates both a more economic change of direction and a more efficient acceleration. Our subjects were not evaluated on speed, but the literature confirms that PT improves it [17,51], and is logical that when acceleration after turning around a cone improves, the time necessary to reach the next cone will decrease. Furthermore, at the moment it is already documented that PT improves the speed of step initiation, thereby contributing to the decrease of contact time [51,56,57] and rate force development [47,49,52], resulting in a better capacity to accelerate.

### 4.3. Dribbling Effects

The present study confirms that PT improves the dribbling performance too (Table 3). The improvement in the dribbling performance was similar to that found for agility, in fact an agility test course that subjects had to cover it carrying the ball.

Sports specific skills were not studied extensively in the presence of PT impact. One author [30], found that a 12 weeks PT intervention improved the passing skills in basketball players, whereas two others [32,33] involved the dribbling ability of soccer players. The test course in the experiment of Bekris et al. [32], was more complex than ours, being covered by the players in 19–20 s; with a nonsignificant alteration after 10 weeks of PT intervention. In a simpler test [33], with only three poles within a 10m course, the 10 to 12 years old players displayed a significant improvement of 0.3 and 0.8 s, after performing a complex proprioceptive-coordinative training for 6 and 12 months, respectively.

Other soccer skills were investigated and showed improvement following PT: Jug 200, Jug body 1, Jug body 2, long and short passing [32], ball velocity [33,34], and landing the ball on a 2 × 2 m sector from 10m distance [33]. Apart from the factors and mechanisms presented in relation with PT effect on agility, the improvement in SDT could be explained by some alterations in the biomechanics of dribbling. Very recently it was demonstrated that the superiority of faster players in dribbling consists of the ability to run with the ball through a shorter path in a more economical way, as a result of higher foot-to-ball contacts, decreased mediolateral and vertical center of mass deviations, higher right stride cadence with lower variability, and reduced hip and knee flexion range of motion [58]. The respective study did not address PT indeed, but it cannot be excluded that some of the above parameters could have been altered after intervention in our study. The exercises included in the second subprogram may have contributed the most to this dribbling improvement, as they involved ball manipulation in addition to their balance assignment, a dual-task that has been shown to be more physiologically demanding in even simpler performances such as walking [59,60]. However, further research is needed to verify these suppositions.

The strength points of this study were the following: the participation of junior soccer players who have been playing soccer for more than 4 years; the employment of two surface types, firm and foam, for the assessment of balance; the high number of tests administered in order to assess balance, agility and lower limb strength; the design and the implementation of the proprioceptive training program with exercises adapted to the participants’ age and the characteristics of the soccer play; the ideation of the program so that it combined exercises with and without the ball. The drawbacks of the study are: the limitation of the subjects to 14 years old junior players; the lack of inclusion of a sample of female players and, eventually, a comparison of the results obtained by the male and the female players’ sample.

## 5. Conclusions

The results of the present study suggest that soccer players of around 14 years of age who have carried out the PT program aiming to improve balance and explosive strength, together with agility and dribbling capacity have had superior results compared to the control group. Analyzing teenage soccer players, we have demonstrated the efficiency of proprioceptive training in this period of pre-adulthood, when balance is easily affected by the particularities of growth and somatic development. The analysis of our findings shows that the level of balance is influenced by the type of supporting surface, so that the more elastic the surface is, the more involved are the capacity of balance and the sense of posture control.

The findings of our study support the idea that, through the design and implementation of a PT program, the level of development of physical fitness can be improved, together with the enhancement of performance for soccer specific abilities. Such programs should be used by coaches as a component of the usual soccer training program to maximize the acquisition of abilities before reaching the age of performance and to maximize sport performance. The impact of the implementation of a PT program depends on the characteristics of the players’ age, their level of preparation and the specific traits of the soccer game. However, further research is still needed, especially to confirm the soccer-specific effects.

## Figures and Tables

**Table 1:** Pre- and post-test of the intervention balance performances (errors)-BESS.

Tests	Types of Surfaces	Group	Phase of Test	X ± SD	DX	95% CI Lower	95% CI Upper	t	p	d
Single-leg stance	On firm surface	Control	Pre-test	0.911 ± 0.592	0.16	−0.006	0.181	0.235	0.157	0.258
			Post-test	0.752 ± 0.637						
		Experiment	Pre-test	1.084 ± 1.144	0.68	−0.024	0.212	0.102	0.006	0.731
			Post-test	0.406 ± 0.642						
	On foam surface	Control	Pre-test	3.582 ± 1.264	0.281	−0.137	0.462	0.712	0.218	0.273
			Post-test	3.251 ± 1.152						
		Experiment	Pre-test	3.665 ±2.938	1.904	−1.193	2.782	0.587	0.002	0.713
			Post-test	1.561 ± 2.956						
Double-leg stance	On firm surface	Control	Pre-test	0.413 ± 0.076	0.011	−0.005	0.324	0.326	0.282	0.179
			Post-test	0.402 ± 0.042						
		Experiment	Pre-test	0.466 ± 0.428	0.264	−0.172	0.486	0.117	0.000	0.670
			Post-test	0.202 ± 0.356						
	On foam surface	Control	Pre-test	2.325 ± 0.987	0.112	−0.092	0.723	0.825	0.006	0.122
			Post-test	2.213 ± 0.839						
		Experiment	Pre-test	2.427 ± 1.942	1.288	−0.231	2.298	0.246	0.000	0.696
			Post-test	1.139 ± 0.251						
Tandem stance	On firm surface	Control	Pre-test	1.082 ± 0.286	0.101	−0.068	0.186	0.214	0.072	0.211
			Post-test	0.981 ± 0.451						
		Experiment	Pre-test	1.167 ± 0.938	0.284	−0.108	0.492	0.081	0.000	0.752
			Post-test	0.451 ± 0.965						
	On foam surface	Control	Pre-test	1.037 ± 0.301	0.066	−0.351	0.226	0.357	0.196	0.208
			Post-test	0.971 ± 0.331						
		Experiment	Pre-test	1.389 ± 1.499	0.778	−0.405	0.926	0.127	0.000	0.617
			Post-test	0.611 ± 0.965						
Total BESS score	Both surfaces (firm + foam)	Control	Pre-test	9.350 ± 2.114	0.780	−0.506	1.158	0.475	0.246	0.380
			Post-test	8.570 ± 1.987						
		Experiment	Pre-test	10.198 ± 8.987	5.828	−4.372	6.316	0.102	0.000	0.705
			Post-test	4.370 ± 7.453						

X ± DS—mean ± standard deviation, DX—difference of means, 95% CI Lower/Upper—confidence interval with two level lower and upper, t—Student’s *t*-test, p—statistical level of probability, d—Cohen ‘d effect size.

**Table 2:** Pre- and post-intervention jump performances (cm).

Tests	Group	Phase of Test	Leg	X ± SD	DX	95% CI Lower	95% CI Upper	t	p	d
Single-leg forward jump	Control	Pre-test	R	160.000 ± 9.957	−1.083	6.911	4.744	−0.374	0.710	0.109
		Post-test	R	161.083 ± 9.896						
		Pre-test	L	155.833 ± 8.597	−0.583	−5.704	4.537	−0.229	0.820	0.066
		Post-test	L	156.416 ± 8.989						
	Experiment	Pre-test	R	159.166 ± 4.793	−3.916	−4.950	−2.882	−7.622	0.000	0.693
		Post-test	R	163.083 ± 4.493						
		Pre-test	L	157.500 ± 7.811	−2.416	−3.088	−1.744	−7.236	0.000	0.322
		Post-test	L	159.916 ± 7.175						
Single-leg lateral jump	Control	Pre-test	R	128.166 ± 8.513	−0.333	−4.984	4.317	−0.144	0.886	0.039
		Post-test	R	128.500 ± 8.460						
		Pre-test	L	125.750 ± 6.239	−0.583	−6.230	5.064	−0.208	0.836	0.088
		Post-test	L	126.333 ± 6.909						
	Experiment	Pre-test	R	127.500 ± 5.780	−2.937	−5.325	−0.549	−2.475	0.017	0.529
		Post-test	R	130.437 ± 5.297						
		Pre-test	L	125.125 ± 6.159	−2.791	−4.614	−0.968	−3.081	0.003	0.447
		Post-test	L	127.916 ± 6.327						
Single-leg vertical jump	Control	Pre-test	R	19.252 ± 1.868	−0.306	−0.983	0.371	−0.909	0.368	0.169
		Post-test	R	19.558 ± 1.732						
		Pre-test	L	19.066 ± 2.191	−0.247	−1.305	0.809	−0.472	0.639	0.121
		Post-test	L	19.314 ± 1.896						
	Experiment	Pre-test	R	19.650 ± 2.996	−1.352	−2.295	−0.408	−2.883	0.006	0.584
		Post-test	R	21.002 ± 2.266						
		Pre-test	L	19.450 ± 1.467	−0.964	−1.564	−0.364	−3.234	0.002	0.523
		Post-test	L	20.414 ± 2.151						
Double-leg CMJ	Control	Pre-test	-	35.300 ± 2.380	−0.516	−1.391	0.358	−1.188	0.241	0.200
		Post-test	-	35.816 ± 2.758						
	Experiment	Pre-test	-	35.716 ± 2.801	−1.775	−2.516	−1.033	−4.817	0.000	0.661
		Post-test	-	37.491 ± 2.563						

X ± DS—mean ± standard deviation, DX—difference of means, R = right; L = left; CMJ = countermovement jump, t—Student’s *t*-test, p—statistical level of probability, d—Cohen ‘d effect size.

**Table 3:** Pre- and post-intervention agility and dribbling performances (sec.).

Tests	Group	Phase of Test	X ± SD	DX	95% CI Lower	95% CI Upper	t	p	d
Agility right side	Control	Pre-test	8.442 ± 0.592	0.120	−0.002	0.242	1.970	0.055	0.205
		Post-test	8.322 ± 0.578						
	Experiment	Pre-test	8.445 ± 0.426	0.402	0.293	0.511	7.435	0.000	0.747
		Post-test	8.043 ± 0.449						
Agility left side	Control	Pre-test	8.680 ± 0.620	0.086	−0.003	0.176	1.933	0.059	0.144
		Post-test	8.593 ± 0.580						
	Experiment	Pre-test	8.655 ± 0.415	0.319	0.264	0.374	11.694	0.000	0.658
		Post-test	8.335 ± 0.548						
Agility right + left side	Control	Pre-test	17.122 ± 0.447	0.054	−0.114	0.224	0.649	0.519	0.121
		Post-test	17.067 ± 0.457						
	Experiment	Pre-test	17.111 ± 0.443	0.240	0.083	0.397	3.078	0.003	0.508
		Post-test	16.871 ± 0.499						
Short dribbling test	Control	Pre-test	13.630 ± 0.769	0.099	−0.192	0.391	0.686	0.496	0.129
		Post-test	13.530 ± 0.777						
	Experiment	Pre-test	13.575 ± 0.514	0.273	0.162	0.384	4.956	0.000	0.513
		Post-test	13.302 ± 0.548						

X ± DS—mean ± standard deviation, DX—difference of means, t—Student’s *t*-test, p—statistical level of probability, d—Cohen ‘d effect size.
